# Notes and Notices

**Published:** 1893-02

**Authors:** 


### NOTES AND NOTICES.

Dr. M. D. Satterlee has removed from Hartstown, Penna., to 1233 Lexington Avenue, Cleveland, Ohio.

Correction.—In the January number, at page 19, line twenty-two from the top for S. L., read Syphilinum$^{mm}$.

In addition to its popularity as an infant food, Malted Milk is now acknowledged to be one of the best diets for Typhoid Fever and many other wasting diseases that has yet been produced. It is the ideal milk diet—very palatable and easily assimilated; more nutritious than beef extracts and superior to raw milk, as it will not form into large hard curds in the stomach. This important feature is obtained by partial digestion of the milk albumen by plant pepsin. Physicians appreciate the vital importance of diet in Typhoid Fever and other cases should give it a trial.

Samples supplied by the manufacturers, Malted Milk Co., Racine, Wis.

During the cold weather physicians, and especially country physicians, will find it very convenient to have a package of Horlick's Malted Milk at home, so that they can make themselves a warm and nourishing drink at a moment's notice when called out for a long drive or to use as a lunch at other times when there is no oppportunity of enjoying a full meal. It is not a stimulant but supplies warm and concentrated nutrition in an easily assimilable form. The Malted Milk Company offer trial packages for this purpose on application.

Announcement.

E. B. Treat, publisher, New York, has in press for early publication, the 1893 International Medical Annual; being the Eleventh yearly issue of this extremely useful work.

A glance at the prospectus gives promises that the 1893 issue will be better than any of its predecessors.

There are thirty-eight distinguished specialists on its corps of editors, carefully selected from among the most eminent Physicians and Surgeons of America, England, and the Continent.

It arranges, in a practical way for ready reference, what is worth preserving of the year's medical literature, together with a number of important papers specially written; and will contain over 6,000 references to diseases and their remedies; many illustrations in black and colors being used where helpful in explaining the text.

The service rendered by this work, giving the year's progress in Medicine and Surgery so conveniently and at so low a price ($2.75), cannot be overestimated.

Altogether it makes a most desirable, if not an absolutely necessary, investment for the practitioner.

Peroxide of Hydrogen.—Mr. Charles Marchand, proprietor of Marchand's Peroxide of Hydrogen, has issued an open letter to Dr. Jacobi of New York, who has been attacking the Peroxide preparation, and classing the proprietor among "quacks and patent-medicine venders." Dr. Jacobi intimates that it is possible to do harm with peroxide of hydrogen, and that it may even cause diphtheria. If this latter assertion be true, then it must often radically cure cases of diphtheria by reason of its being the simillimum. Thus Dr. Jacobi's assertion would constitute a good argument to homœopathists for its use in such cases and its careful proving by our school.

THE MIDWAY SANITARIUM for Consumptives has lately been started at Larned, Kansas, by a homœopathic physician, Dr. G. J. Waggoner. It is situated, as its name denotes, midway between the mountains and the low lands, at an altitude of three thousand feet above the sea. Here people afflicted with consumption and other lung troubles can go for change of climate and at the same time be subjected to homœopathic treatment.

Send for a circular to Dr. G. J. Waggoner, Midway Sanitarium, Larned, Pawnee County, Kansas.

THE YALE CHAIR.

St. Louis, Mo., July 26th, 1891.

I now am the owner of a Yale Chair, and I do not see how there could be anything more perfect than it is. I will be glad to recommend the Yale to any one that stands in need of a chair. H. L. HENDERSON, M. D.

CHILDHOOD.—Mothers and teachers are talked to pretty often upon the subject of their duties, while fathers escape sermonizing. But Mrs. Kate Tannatt Woods has her eye upon these busy lords of creation, whose place as parent is a sinecure, and she addresses some pertinent suggestions to them in the December number of *Childhood.* They will have to take her womanly scolding in good part, for how can they deny such a remark, as, for instance, the following: "A woman who really loves her husband and children, finds all service for them a pleasure, and is frequently inclined to foster selfishness in men and boys. The masculine portion of humanity, after long years of petting and continuous ministration from women, have reached a point where receiving is more natural than giving."

THE SCIENTIFIC AMERICAN, published by Munn & Co., New York, presents weekly to its readers the best and most reliable record of various improvements in machinery, while the scientific progress of the country can in no way be gleaned so well as by the regular perusal of its pages.

"THE NEW YORK WORLD'S" GREATEST YEAR.—The year just closed was the most prosperous in the history of *The World.* The total circulation for the year was 139,262,685. The increase over the total circulation in 1891 was 23,724,860, an average gain of 63,958 per day. This gain alone in the average circulation per day, during the year, exceeds the circulation per day of a majority of the newspapers in New York. The average circulation per day of *The World* for the year was 380,499—a figure unapproached by any other newspaper printed in the English language.

